# The Relationships of Health Behaviour and Psychological Characteristics with Spontaneous Preterm Birth in Nulliparous Women

**DOI:** 10.1007/s10995-016-2160-4

**Published:** 2016-08-31

**Authors:** Ruth Baron, Saskia J. te Velde, Martijn W. Heymans, Trudy Klomp, Eileen K. Hutton, Johannes Brug

**Affiliations:** 1 Department of Midwifery Science, Midwifery Academy Amsterdam Groningen (AVAG) and the EMGO Institute for Health and Care Research, VU University Medical Centre, P.O. Box 7057, 1007 MB Amsterdam, The Netherlands; 2 Department of Epidemiology and Biostatistics and the EMGO Institute for Health and Care Research, VU University Medical Centre, P.O. Box 7057, 1007 MB Amsterdam, The Netherlands; 3 Department of Methodology and Applied Biostatistics, Faculty of Earth and Life Sciences, VU University Amsterdam, De Boelelaan 1085, 1081 HV Amsterdam, The Netherlands; 4 Department of Obstetrics and Gynecology, McMaster University, 1280 Main Street West, MDCL 2210, Hamilton, ON L8S 4K1 Canada

**Keywords:** Preterm birth, Primary care, Maternal health behaviours, Health control beliefs

## Abstract

*Objectives* Preterm birth is the leading pregnancy outcome associated with perinatal morbidity and mortality and remains difficult to prevent. There is evidence that some modifiable maternal health characteristics may influence the risk of preterm birth. Our aim was to investigate the relationships of self-reported maternal health behaviour and psychological characteristics in nulliparous women with spontaneous preterm birth in prenatal primary care. *Methods* The data of our prospective study was obtained from the nationwide DELIVER multicentre cohort study (September 2009–March 2011), which was designed to examine perinatal primary care in the Netherlands. In our study, consisting of 2768 nulliparous women, we estimated the relationships of various self-reported health behaviours (smoking, alcohol consumption, folic acid supplementation, daily fruit, daily fresh vegetables, daily hot meal and daily breakfast consumption) and psychological characteristics (anxious/depressed mood and health control beliefs) with spontaneous preterm birth as a dichotomous outcome. Due to the clustering of clients within midwife practices, Generalized Estimating Equations was used for these analyses. *Results* Low health control beliefs was the sole characteristic significantly associated with spontaneous preterm birth (odds ratio 2.26; 95 % confidence interval 1.51, 3.39) after being adjusted for socio-demographics, anthropometrics and the remaining health behaviour and psychological characteristics. The other characteristics were not significantly associated with spontaneous preterm birth. *Conclusions for Practice* Maternal low health control beliefs need to be explored further as a possible marker for women at risk for preterm birth, and as a potentially modifiable characteristic to be used in interventions which are designed to reduce the risk of spontaneous preterm birth.

## Significance

*What is already known on this subject?* Spontaneous preterm birth is associated with perinatal mortality and morbidity, but remains difficult to predict and prevent. There is some evidence that maternal health behaviour and psychological characteristics during pregnancy may be associated with spontaneous preterm birth.

*What this study adds*? Of all the self-reported maternal health behaviour and psychological characteristics examined in this study, low health control beliefs was the sole maternal characteristic associated with having a spontaneous preterm birth in nulliparous women. Further studies should explore the role of low health control beliefs as a possible marker, or as a potentially modifiable characteristic to help reduce the risk of spontaneous preterm birth.

## Introduction

Preterm birth occurs in about 7.6 % of all pregnancies in the Netherlands (Stichting Perinatale Registratie Nederland 2013) and is the leading pregnancy outcome associated with perinatal morbidity and mortality (McIntire and Leveno 2008), as well as with physical and mental disabilities later in life (Crump 2015; Loe et al. 2011). Preterm births can either result from spontaneous labour (40–45 % of all preterm births), follow prelabour rupture of membranes (pPROMs) (25–30 %), or be medically indicated (20–25 %) (Goldenberg et al. 2008). The precise etiology of preterm birth is still unknown, but factors found to be associated with preterm birth include infections, cervical anomalies, the extremes of maternal age and being part of disadvantaged populations (Moutquin 2003). Health behaviours and psychological factors, such as smoking (Ion and Bernal 2014), lack of folic acid supplementation (Li et al. 2014), alcohol consumption (O’Leary 2012) and maternal stress and depression (Grote et al. 2010; Vrekoussis et al. 2010) have been found by some studies to be associated with preterm birth. Healthy diets (including fruit, vegetable and fish consumption) have been associated with a lower chance of preterm birth (Englund-Ogge et al. 2014; Leventakou et al. 2014).Women who have had preterm births have also consistently been found to be at an increased risk of cardiovascular diseases themselves, suggesting the presence of similar underlying biological mechanisms and risk factors (Catov et al. 2007; Robbins et al. 2014). Other studies have not found convincing associations with health behaviour characteristics, however (Mutsaerts et al. 2014; Savitz et al. 2012). It is still unclear the extent to which preterm birth could be prevented through health behaviour modifications. Medical interventions to prevent preterm birth have been minimally successful as well, however, and it is possible that preventive measures in primary care, focusing on relevant health behaviours and factors related to lower socio-economic status may turn out to be more effective (Wisanskoonwong et al. 2011). To develop primary care interventions, it is important to examine the relationship of various potentially modifiable factors, such as health behaviour and psychological characteristics with preterm birth.

In the Netherlands, 84.9 % of all pregnant women start their pregnancies under the care of midwives in primary care, as they are considered to be at low risk for pregnancy complications. During pregnancy about one-third (34.7 % in 2013) of women are referred to secondary care as complications arise (Stichting Perinatale Registratie Nederland 2013). This division makes it possible to research pregnancy outcomes in pregnant women, who have no identified risks for adverse pregnancy outcomes at the outset of pregnancy. In the general Dutch population it was found in a period of 8 years (2000–2007) that 6 % of all singleton pregnancies were preterm (1.7 % medically induced, 0.9 % pPROM premature births and 3.4 % spontaneous without pPROM) (Schaaf et al. 2011); it is unknown what proportion of nulliparous women starting out their pregnancies in primary care experience spontaneous preterm birth.

This study aims to describe the prevalence rates of singleton spontaneous preterm births in nulliparous pregnant women starting their pregnancy in primary care, and to investigate the relationships of various health behaviour and psychological factors with spontaneous preterm birth (with or without pPROM) among nulliparous women.

## Methods

Our study is prospective in design and uses data on health behaviour and psychological characteristics collected by the multicentre prospective cohort DELIVER study (September 2009–March 2011), by means of self-administered questionnaires completed by pregnant women in primary prenatal care. DELIVER is an acronym for Data EersteLIjns VERloskunde, which is translated as Data Primary Care Midwifery. This data was linked to the National Midwifery Registry [Landelijke Verloskunde Registratie (LVR1)] and pregnancy record data provided by midwives, for additional information on birth and pregnancy outcomes.

### Recruitment and Study Population

The DELIVER study consisted of 7865 low risk pregnant women starting their pregnancy in primary care. Details on the study can be obtained elsewhere (Mannien et al. 2012). Briefly, 20 different midwifery practices, stratified by region (North, East, South and West), urbanisation level (urban or rural) and practice type (dual or group practice) took part in the study by inviting all their clients to complete three questionnaires during the period of 1 year. To participate, their clients had to understand Dutch, English, Turkish, Berber or Arabic. Written reminders were sent to non-responders and if they had not responded within 1 week, telephone calls were then made by research assistants. Interviews were offered by telephone to Arabic, Berber and Turkish speaking women who had not responded to the initial invitation. The three questionnaires to be completed (one before 35 weeks of pregnancy; one between 35 weeks and birth; one after giving birth) contained many items pertaining to personal experiences with their pregnancy, health (behaviours) and their health care providers. The overall net response of women who had completed at least one of the three questionnaires was 62 % of those who had been invited to participate.

For this study, we used data from the first questionnaire (before 35 weeks of pregnancy) as well as available LVR1 and pregnancy record data. As previous preterm birth is a well-established risk factor for having a preterm birth in multiparous women (Hammond et al. 2013), we chose to focus on spontaneous preterm birth in a population of low risk nulliparous women with singleton pregnancies. Women with medically induced preterm births (as far as that information was available) were excluded. Planned caesarean sections were not excluded due to the fact the LVR1 data does not distinguish between planned and unplanned caesarean sections. The final study population contained 2768 respondents.

### Study Measures

The dependent variable ‘preterm birth’ was calculated using the expected date of birth and the actual date of birth of the child, as recorded by midwives and registered in the National Midwifery Registry (LVR1). Where information was incomplete or missing, this variable was complemented by self-reported information provided by women who had completed the third questionnaire as well (after birth). This variable was dichotomised into ‘full term’ (gestational age at birth of 37 weeks or more) and ‘preterm’ (gestational age at birth of <37 weeks).

#### Independent Variables

Selected health behaviour and psychological characteristics were based on earlier studies and on plausible associations with spontaneous preterm birth. These were folic acid supplementation, alcohol consumption, smoking, daily fruit, daily fresh vegetables, daily hot meal and breakfast consumption, health control beliefs and anxious or depressed mood (Grote et al. 2010; Herrmann et al. 2001; Ion and Bernal 2014; Li et al. 2014; Myhre et al. 2013; O’Leary 2012; Vrekoussis et al. 2010).

Health behaviour characteristics: Respondents were asked if they had taken folic acid for this pregnancy with response options ‘yes’ or ‘no’ and whether they had consumed any alcohol since knowing they were pregnant, with response options ‘yes’ or ‘no’. They were asked to report their current smoking status, with three response options ‘daily smoker’, ‘occasional smoker’ or ‘not at all’. Daily smokers were asked to indicate the average number of cigarettes they smoked daily and occasional smokers were asked to indicate the average number of cigarettes they smoked weekly. This led to a composite variable based on the mean number of cigarettes smoked daily, with the categories ‘non-smoker’, ‘light smoker’ (<10 cigarettes daily) and ‘heavy smoker’ (≥10 cigarettes daily). Respondents were also asked if they ate fruit daily, fresh vegetables daily and a hot meal daily, with all three questions containing response options ‘yes’ or ‘no’. The variable ‘breakfast consumption’ was obtained by an item asking women how often per week they ate breakfast; the four response options were categorized into ‘daily’ and ‘4–6 times per week’ and ‘up to 3 times per week’.

Psychological characteristics: The variable health control beliefs was included, as feeling in control is often considered a component of managing stress (Tragea et al. 2014). This variable was obtained from an item asking respondents to what extent they believed they could control their health with their own behaviours. This item was designed to measure internal health locus of control and is similar to the statement ‘The main thing that affects my health is what I myself do’ in the Health Locus of Control scales developed by Wallston et al. (1978). The four possible response options were dichotomized into ‘quite a bit/very much’ and ‘very little/not at all’. Another psychological variable for this study ‘anxious or depressed mood’ was an item obtained from the EuroQol questionnaire (EuroQol Group 1990) asking respondents about their current mood, and containing three response options ‘not at all anxious or depressed’, ‘somewhat anxious or depressed’ and ‘very anxious or depressed’. The response options were dichotomized into ‘not at all anxious or depressed’ and ‘somewhat/very anxious or depressed’.

#### Potential Confounders

Possible confounders, based on earlier research, were partner status (Lopez and Breart 2013), high (>35 years) and low (<20 years) maternal age (Ip et al. 2010), low maternal education and being of ethnic minority (Goldenberg et al. 2008). Potential anthropometric confounders are maternal underweight and overweight (Torloni et al. 2009) and lower maternal height (different studies use various categories and cut-off points ranging from <150 to 170 cm) (Han et al. 2012).

##### Socio-demographics

Women were asked to report their age based on data of birth and this was categorized as ‘<25 years’, ‘25–35 years’, and ‘>35 years’. They were also asked their highest attained educational level which was then categorized as either ‘lower’ (lower vocational education or less), ‘medium’ (secondary school, or mid-level vocational education) or ‘higher’ (college, university or post-graduate education). Respondents were asked about their country of birth as well as their parents’ country or countries of birth and the ethnicity variable was categorized as either ‘Dutch’ or ‘non-Dutch ethnicity’, based on the definition used by Statistics Netherlands (Statistics Netherlands, Consulted January 2015). If both parents were born in the Netherlands, they are considered Dutch and if at least one of their parents was born in another country, they are considered to be of non-Dutch ethnicity. Women were asked if they have a spouse or partner with dichotomous response options ‘yes’ or ‘no’.

##### Anthropometrics

Respondents were asked to report their height in centimetres and weight in kilograms before they became pregnant. We categorized the variable ‘height’ as ‘>177 cm’, ‘164–177 cm’ and ‘<164 cm’. The variable Body Mass Index (BMI) was calculated using maternal height and weight and categorized according to the World Health Organization criteria of weight status: ‘underweight’ (<18.5 kg/m^2^), ‘normal weight’ (18.5–24.99 kg/m^2^), ‘overweight’ (25–29.99 kg/m^2^) and ‘obesity’ (30+ kg/m^2^).

### Statistical Analyses

Frequencies and means of socio-demographics and anthropometrics were calculated to portray the characteristics of the study population as a whole. Missing data analyses showed that 27.3 % of cases had missing data in at least one of the study variables, with the variable ‘preterm/full term birth’ having 20.8 % missing data, followed by BMI which had 5.8 % missing data. We examined the variables with missing data by testing them for associations with other variables using multiple logistic regression. In these models, the variables with missing data (yes/no) were treated as outcome. These analyses showed that the missing data of ‘preterm/full term birth’ was associated with higher educational level and lower maternal age, and the missing data of BMI with lower maternal education. These associations meant there was an increased likelihood that the data were of the type Missing At Random (MAR)(White et al. 2011). Multiple imputation was therefore carried out using all the variables in our study in the imputation model. We generated a dataset containing 27 new datasets (due to the 27.3 % of cases with missing data).

As our study consisted of two levels of data, midwife practices and individual pregnant women, we used Generalized Estimating Equations (GEE) to adjust for possible correlations within each midwife practice. First the associations of social demographics and anthropometrics (considered as confounders) with preterm birth were examined univariably using GEE logistic regression. Then the association of each health behaviour and psychological characteristic with preterm birth was examined univariably using GEE logistic regression in model 1. Each characteristic was then examined again, but with adjustment for potential confounders in stages: first socio-demographics in model 2, then socio-demographics and anthropometrics in model 3, and finally socio-demographics, anthropometrics and all other health behaviour and psychological characteristics together in model 4. The GEE analyses on the multiple imputed datasets resulted in pooled regression coefficients and 95 % intervals of regression coefficients. Odds ratios with their 95 % confidence intervals were calculated from these coefficients and were reported for each model. Sensitivity analyses were carried out to compare complete case analyses with multiple imputed data analyses and to compare spontaneous preterm with all types of preterm birth as outcome; differences and similarity in results were reported. All analyses were carried out in IBM SPSS version 22.

## Results

Spontaneous preterm birth occurred in 138/2196 (6.3 %) pregnancies in our nulliparous population of complete cases, of which the median gestational age at birth was 35.1 weeks (Table 1). The average age of our nulliparous population was 28.8 (SD 4.5), 50.6 % were highly educated and 16.1 % of women were of non-Dutch ethnicity. The median number of weeks of pregnancy at the time of questionnaire completion in the population of complete cases was 20 for the full term births and 20 for preterm births. The socio-demographic and anthropometric characteristics (considered to be potential confounders) which had a significant relationship with having a spontaneous preterm birth were lower educational level and lower maternal height (<164 cm).

**Table 1:** Proportions of socio-demographics and anthropometrics of nulliparous women by total births, and odds ratios (OR) + 95 % confidence intervals (CI) of univariable relationships between socio-demographics/anthropometrics and spontaneous preterm birth, using Generalized Estimating Equations (GEE)

Socio demographics and anthropometrics of nulliparous women	Total (all births) N/2768 (%)	Spontaneous preterm births Univariable^a^ OR [95 % CI]
*Gestational age at birth*		
Median	39.9	35.1
Range	21.1–42.6	21.1–36.9
Missing	572	
*Age in years*		
Mean (SD)	28.8 (4.5)	
25–35	2098 (75.9)	1
<25	453 (16.4)	1.36 [0.88, 2.10]
Above 35	213 (7.7)	1.54 [0.84, 2.80]
Missing	4	
*Spouse/partner*		
Yes	2707 (98.0)	1
No	55 (2.0)	1.18 [0.45, 3.10]
Missing	6	
*Education*		
High	1398 (50.6)	1
Medium	996 (36.0)	1.20 [0.91, 1.60]
Low	370 (13.4)	**1.72 [1.14, 2.58]**
Missing	4	
*Ethnicity*		
Dutch	2315 (83.9)	1
Non-Dutch	443 (16.1)	1.23 [0.77, 1.97]
Missing	10	
*Height*		
>177 cm	420 (15.4)	1
164–177 cm	1880 (68.9)	1.53 [0.84, 2.79]
<164 cm	428 (15.7)	**2.63 [1.42, 4.87]**
Missing	40	
*BMI*		
Normal	1809 (69.4)	1
Underweight	87 (3.3)	1.69 [0.94, 3.06]
Overweight	518 (19.9)	0.84 [0.54, 1.29]
Obese	194 (7.4)	0.63 [0.29, 1.38]
Missing	160	

Bold: significant

^a^Odds ratios and 95 % CI based on multiple imputed data

Univariable analyses (Table 2) showed heavy smoking (10+ cigarettes per day) and low health control beliefs to be significantly associated with preterm birth. Heavy smoking was no longer significant, when corrected for socio-demographic characteristics. Low health control beliefs retained its significance [odds ratio (OR) 2.26; 95 % confidence interval (CI) 1.51, 3.39] after being corrected for socio-demographics, anthropometrics, the remaining health behaviour characteristics and the psychological characteristic ‘depression or anxiety’. All the other characteristics (folic acid supplementation, alcohol consumption, daily fruit, daily fresh vegetables, daily breakfast, daily hot meal consumption, depression or anxiety) were not significantly associated with spontaneous preterm birth.

**Table 2:** Odds ratios (OR) and 95 % confidence intervals (CI) showing the relationships of various health behaviour and psychological characteristics with spontaneous (sp) preterm birth in nulliparous women, adjusted for socio-demographics, anthropometrics and other health behaviour/psychological characteristics using Generalized Estimating Equations (GEE) after multiple imputation

Health behaviour and psychological characteristics of nulliparous women	Sp preterm birth	Model 1 Univariable	Model 2 Multivariable	Model 3 Multivariable	Model 4 Multivariable
	N (%)^a^	OR [95 % CI]	OR [95 % CI]	OR [95 % CI]	OR [95 % CI]
*Smoking*					
No	119/1967 (6.0)	1	1	1	1
Yes, <10 daily	11/165 (6.7)	1.07 [0.57, 2.00]	0.92 [0.47, 1.77]	0.87 [0.45, 1.68]	0.81 [0.41, 1.58]
Yes, ≥10 daily	6/43 (14.0)	**2.44 [1.11, 5.37]**	2.01 [0.84, 4.76]	1.88 [0.81, 4.37]	1.83 [0.78, 4.29]
Missing	27				
*Folic acid supplement*					
Yes	131/2058 (6.4)	1	1	1	1
No	6/129 (4.7)	0.77 [0.38, 1.58]	0.63 [0.30, 1.35]	0.60 [0.28, 1.29]	0.49 [0.22, 1.10]
Missing	11				
*Alcohol consumption*					
No	124/1970 (6.3)	1	1	1	1
Yes	13/213 (6.1)	0.98 [0.57, 1.68]	0.97 [0.57, 1.66]	0.95 [0.55, 1.62]	0.92 [0.53, 1.60]
Missing	17				
*Daily fruit*					
Yes	111/1887 (5.9)	1	1	1	1
No	26/296 (8.8)	1.51 [0.94, 2.41]	1.43 [0.89, 2.32]	1.46 [0.91, 2.36]	1.44 [0.88, 2.36]
Missing	16				
*Daily fresh vegetables*					
Yes	105/1702 (6.2)	1	1	1	1
No	31/481 (6.4)	1.07 [0.72, 1.61]	1.07 [0.72, 1.59]	1.09 [0.73, 1.64]	1.01 [0.66, 1.54]
Missing	17				
*Daily hot meal*					
Yes	131/2098 (6.2)	1	1	1	1
No	6/86 (7.0)	1.18 [0.45, 3.10]	1.14 [0.44, 2.95]	1.18 [0.45, 3.09]	1.11 [0.41, 2.99]
Missing	15				
*Breakfast consumption*					
Daily	116/1902 (6.1)	1	1	1	1
4–6 times p/w	14/191 (7.3)	1.25 [0.70, 2.23]	1.15 [0.64, 2.08]	1.12 [0.63, 1.98]	1.10 [0.60, 2.01]
Up to 3 times p/w	7/90 (7.8)	1.42 [0.73, 2.76]	1.28 [0.66, 2.46]	1.26 [0.65, 2.44]	1.18 [0.60, 2.32]
Missing	16				
*Anxious/depressed mood*					
Not at all	104/1763 (5.9)	1	1	1	1
A little/very much	34/425 (8.0)	1.42 [0.92, 2.21]	1.33 [0.85,2.07]	1.33 [0.84, 2.08]	1.32 [0.82, 2.12]
Missing	11				
*Health control beliefs*					
Very much/quite a bit	99/1845 (5.4)	1	1	1	1
Very little/not at all	39/341 (11.4)	**2.32 [1.61, 3.33]**	**2.22 [1.47, 3.36]**	**2.19 [1.45, 3.31]**	**2.26 [1.51, 3.39]**
Missing	14				

Model 1: univariable (unadjusted)

Model 2: adjusted for socio-demographics (age, education, ethnicity and relationship status)

Model 3: model 2 + adjusted for anthropometrics (BMI and height)

Model 4: model 3 + adjusted for all other health behaviour/psychological characteristics in this study

Bold: significant

^a^Frequencies based on original data with missing values

The sensitivity analyses comparing complete case analyses with multiple imputed analyses showed that underweight was significantly associated with spontaneous preterm birth in the univariable complete case analyses, but was not significant in the multiple imputed dataset. The difference in effect was small, however [(OR 1.89; 95 % CI 1.10, 3.26) vs (OR 1.69; 95 % CI 0.94, 3.06)]. For all the other variables, univariable and multivariable analyses in both complete case and multiple imputed datasets, produced similar results and therefore led to the same conclusions. The sensitivity analyses examining all types of preterm birth as outcome showed similarity in all the relationships, except for low education, low maternal height, and low health control beliefs. Although there was an effect, these variables were not significantly associated with aggregated preterm birth in the univariable analyses. Low health control beliefs remained insignificant in all the models with aggregated preterm birth as outcome.

## Discussion

We aimed to study the prevalence of spontaneous preterm births among nulliparous women starting their pregnancy in prenatal primary care, as well as the association of self-reported health behaviour and psychological characteristics with having a spontaneous preterm birth (<37 weeks of gestation at birth). The prevalence of spontaneous birth in our nulliparous population was 6.3 %, which is somewhat higher than what was found for the Dutch national nulliparous singleton population over the years 2000–2007 (6.3 % vs about 5.5 %) (Schaaf et al. 2011). This may be due to not being able to exclude all preterm births with a medical indication in our study population because of incomplete information on medical inductions and planned caesarian sections.

Low health control beliefs was the only variable significantly associated with spontaneous preterm birth in our study, after adjusting for socio-demographics, anthropometrics and the other health behaviour and psychological characteristics. Women with lower health control beliefs were more than twice as likely to have a spontaneous preterm birth than those with higher health control beliefs. Some studies have previously shown a relationship between low maternal health control beliefs and preterm births (Ashford and Rayens 2013; Pichler-Stachl et al. 2011), but these health control beliefs were measured after birth. There are no studies to our knowledge examining this relationship, where health control beliefs are measured before birth. Stress has been found to be related to preterm birth (McDonald et al. 2014; Messer et al. 2005) and increasing a sense of control has been included in interventions helping pregnant women to manage stress (Tragea et al. 2014). Increasing health control beliefs has also found to be effective in reducing negative outcomes such as postnatal depression (Moshki et al. 2014). Group prenatal care, such as CenteringPregnancy™, shows some promise in improving birth outcomes, such as preterm birth and low birth weight (Thielen 2012). In this approach to care, besides providing increased education and extra support from health care givers and peers for pregnant women, self-management is also encouraged by letting women play a role in their own prenatal health care, such as weighing themselves and taking their own blood pressure (Walker and Worrell 2008). It is plausible that this self-management also leads to higher health control beliefs, and played a role in the reduction of preterm births observed in earlier studies. The perception of having control of one’s own health may also be related to the perception of having control over one’s own behaviours. Perceived behavioural control as described in the Theory of Planned Behaviour (Ajzen 1985) and self-efficacy, as described in Social Cognitive Theory (Bandura 1986) have been described as (modifiable) constructs necessary for changing one’s own health behaviours. As this an observational study, we cannot draw conclusions about any causality between low health control beliefs and spontaneous preterm birth. Further studies are needed to investigate whether having low health control beliefs is a marker for other factors related to preterm birth which we could not measure, and whether modifying health control beliefs could somehow lead to more positive pregnancy outcomes.

Heavy smoking was significantly associated with preterm birth in the univariable analyses, but although an effect was still apparent, it was no longer significant after being corrected for socio-demographics. This may be due to the relatively small numbers of heavy smokers and preterm births, as well as the finding that smoking is also associated with lower education (Baron et al. 2015). Earlier studies of smoking and preterm births have had varying results, from no association (Dekker et al. 2012), to associations with very early preterm births (Kyrklund-Blomberg et al. 2005) and medically induced preterm births (Aliyu et al. 2010). Some studies have also suggested that smoking is more responsible for foetal growth restriction than preterm birth (Horta et al. 1997). Data which can clearly separate growth-restricted from non-growth-restricted preterm infants, may shed some more light on the actual pathological effects of smoking.

Although other studies have suggested that diets containing high amounts of certain fruits and vegetables can reduce the risk for preterm birth (Myhre et al. 2013; Smith et al. 2015), our study showed that not consuming fruit daily only bordered in significance of being related to spontaneous birth, and not consuming vegetables daily showed no relationship at all. With the exception of alcohol and folic acid, all of the odds ratios of the more suboptimal health behaviour and psychological characteristics pointed in the direction of an increased risk for spontaneous preterm birth, although none of the odds were very high and none of the relationships significant. Whether the effects of health behaviour and psychological characteristics are really not that strong, or whether our items were not sensitive and precise enough to really capture an actual association, needs to be examined further.

### Strengths and Limitations

Our items were all self-reported, making it possible that some of them, for example smoking, were underreported. The respondents were informed, however, that the information they provided would remain anonymous; this may have helped to make the responses more accurate. When trying to identify markers for preterm birth, self-reported items may be advantageous, as the information is relatively easy for caregivers to ask about.

The health control beliefs variable was an adjusted question based on items developed in earlier scales for measuring internal health locus of control (Rotter 1966; Wallston et al. 1978). Although most health locus of control scales have multiple items, our measurement of health control beliefs consisted of one item. This was due to the necessary restriction of items in the DELIVER questionnaires, which aim was to assess a wide range of aspects relating to maternal health and prenatal care. We cannot conclude, therefore, that the respondents with low and high health control beliefs in our study consistently have low and high health control beliefs in all situations. We believe that this item does provide a good indication of women’s self-perceived control over their own health. Further studies are necessary however, using validated and multidimensional scales of health control beliefs to verify our findings.

The nutrition items in the questionnaire were broadly formulated and the ‘yes’ versus ‘no’ response options possibly too limited to detect any relationship with preterm birth. It would be worthwhile to examine the relationship between nutrition and preterm birth further, by using questionnaire items which ask for information on types, quantity and frequency of specific foods consumed.

Our nulliparous population was more highly educated and had a lower proportion of non-Dutch ethnicity than the general Dutch female population between 15 and 55 years of age (50.6 vs 28.2 and 16.1 vs 22.7 %, respectively) (Statistics Netherlands 2010). This is unlikely, however, to have influenced the strengths of the relationships found between the various health behaviour and psychological characteristics and preterm birth. A strength of our study was the selection of only a nulliparous singleton population in prenatal primary care, which enabled us to have a relatively ‘healthy’ population, free of obstetrical risks and medical issues requiring secondary care. Another strength of our study was that all of the health behaviour and psychological characteristics were measured before birth, limiting recall bias or other bias influenced by the pregnancy outcome itself.

There are advantages and disadvantages to examining spontaneous and indicated preterm births separately, as well as together. Different types of preterm birth share many similar risk factors, such as pre-eclampsia and foetal growth restriction, but the size of the effects of these risk factors differ per preterm birth type (Savitz et al. 2005). Some of the effects were no longer significant in the aggregated preterm birth models in our study, possibly because of contrasting risk factors. Lower education, for instance, has been found to be associated with spontaneous preterm birth and higher education with indicated preterm birth, meaning their effects could cancel each other out, when aggregating preterm birth (Savitz et al. 2005). It is possible that similar patterns are occurring for health control beliefs and type of preterm birth, but this would need further investigation.

### Conclusions

In our study, we found almost no health behaviour and psychological characteristics to be associated with spontaneous preterm birth. Low health control beliefs was the sole variable associated with spontaneous preterm birth after adjusting for potential confounding factors. Maternal low health control beliefs need to be explored further as a possible marker for women at risk for preterm birth and as a potentially modifiable characteristic to be used in interventions which are designed to reduce the risk of spontaneous preterm birth.
